# Identifying biotic drivers of population dynamics in a benthic–pelagic community

**DOI:** 10.1002/ece3.7298

**Published:** 2021-03-28

**Authors:** Louise Forsblom, Andreas Lindén, Jonna Engström‐Öst, Maiju Lehtiniemi, Erik Bonsdorff

**Affiliations:** ^1^ Marine Research Centre Finnish Environment Institute Helsinki Finland; ^2^ Environmental and Marine Biology Åbo Akademi University Turku Finland; ^3^ Natural Resources Institute Finland Helsinki Finland; ^4^ Novia University of Applied Sciences Ekenäs Finland

**Keywords:** Baltic Sea, benthic–pelagic coupling, density dependence, interactions, population dynamics, state‐space model

## Abstract

Benthic species and communities are linked to pelagic zooplankton through life‐stages encompassing both benthic and pelagic habitats and through a mutual dependency on primary producers as a food source. Many zooplankton taxa contribute to the sedimentary system as benthic eggs. Our main aim was to investigate the nature of the population level biotic interactions between and within these two seemingly independent communities, both dependent on the pelagic primary production, while simultaneously accounting for environmental drivers (salinity, temperature, and oxygen conditions). To this end, we applied multivariate autoregressive state‐space models to long (1966–2007) time series of annual abundance data, comparing models with and without interspecific interactions, and models with and without environmental variables included. We were not able to detect any direct coupling between sediment‐dwelling benthic taxa and pelagic copepods and cladocerans on the annual scale, but the most parsimonious model indicated that interactions within the benthic community are important. There were also positive residual correlations between the copepods and cladocerans potentially reflecting the availability of a shared resource or similar seasonal dependence, whereas both groups tended to correlate negatively with the zoobenthic taxa. The most notable single interaction within the benthic community was a tendency for a negative effect of *Limecola balthica* on the amphipods *Monoporeia affinis* and *Pontoporeia femorata* which can help explain the observed decrease in amphipods due to increased competitive interference.

## Graphical abstract

We investigated the abiotic drivers and biotic interactions of pelagic cladocerans and copepods and multiple macrozoobenthic species. Previous laboratory studies have shown that benthic species can reduce zooplankton emergence from resting eggs in the sediment. Our most supported model included no interactions between zooplankton and benthos on interannual scale, but did contain interactions between benthic species.

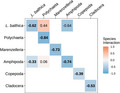

## 1 INTRODUCTION

Marine ecosystems and communities are influenced by climate change through precipitation‐driven salinity decrease in marginal seas, warming, and hypoxia or anoxia (Hoegh‐Guldberg & Bruno, 2010; Kabel et al., 2012; Thompson et al., 2015). Whereas some biological processes related to benthic–pelagic coupling are uncertain and insufficiently quantified, eutrophication and hypoxia are known to have a detrimental effect on, for example, bioturbation, and hence, nutrient and oxygen fluxes to and from the sediment (Karlson et al., 2007; Norkko et al., 2012; Griffiths et al., 2017). When considering long‐term effects of such extrinsic variables, both population‐ and community level processes are important to account for, as they together explain the functioning of the community (Salo et al., 2019). This calls for joint investigation of biotic interactions and extrinsic variables in forming and maintaining community dynamics (Walther, 2010 and references therein). This is particularly important in systems with few species, where a single species can substantially influence the carbon cycling, such as the bivalve *Limecola balthica* in the Baltic Sea (Ehrnsten et al., 2019; Elmgren, 1984; Elmgren & Hill, 1997).

Community composition and interactions influence the energy fluxes in the food webs (Andersson et al., 2015; Karlson et al., 2010), and many benthic taxa depend on sedimentary matter as their primary food source (Eriksson Wiklund & Andersson, 2014). The benthic zone is also an important source of energy for higher trophic levels (Kiljunen et al. 2020). How these organic matter fluxes are affected by mesozooplankton in natural systems is not well quantified, but high numbers of pelagic grazers have the potential to reduce the sedimentation of primary production (Tamelander et al., 2017). The seafloor is also important for survival of several zooplankton taxa due to the production of benthic eggs and their dormancy in the sediments over harsh winter times, especially in shallow coastal areas (Katajisto et al., 1998; Viitasalo, 1992). The zoobenthic and zooplanktonic species are not only linked through their dependency of primary production as a food source, but also because many benthic taxa have planktonic larval stages. In addition, many planktonic taxa spend part of their life cycle as benthic eggs in the sediment (Katajisto et al., 1998). Some benthic species, such as the amphipods *Monoporeia affinis* and the bivalve *Limecola balthica*, have been shown to feed on copepod eggs (*L. balthica)* and thereby negatively influence the emergence of copepods (Karlson & Viitasalo‐Frösen, 2009; Viitasalo et al., 2007). *M. affinis* has been shown to reduce the hatching of *Eurytemora affinis* by 60%–70% (Albertsson & Leonardsson, 2001). However, significant negative interactions are reported between these and other zoobenthic species (Ejdung et al., 2000; Ejdung & Elmgren, 2001), illustrating the complexity of interactions both between the benthic taxa and between the pelagic and the benthic realm.

Multivariate autoregressive models have been used to investigate temporal changes in abundance together with environmental effects and biotic interaction of pelagic and lake systems (Barraquand et al., 2018; Francis et al., 2012, 2014; Griffiths et al., 2016; Ives et al., 2003). These models are especially convenient when the number of species is relatively small and potential causes and directions of biotic interaction effects are well known, as high numbers of species easily lead to high numbers of parameters to estimate and false positive effects can occur (Barraquand et al., 2019; Ives et al., 2003). In that regard, the Baltic Sea with its long history of marine research is an ideal study system with a relatively small number of species due to its short geological history and brackish environment (Elmgren & Hill, 1997; Reusch et al., 2018). Few studies using similar models considering interactions have been conducted in the northern Baltic Sea (but see Griffiths et al., 2016, Torres et al., 2017), and none connecting zoobenthos and zooplankton.

Trends in the major benthic taxa have been previously investigated by Rousi et al. (2013), who noted a marked shift in the benthic community during the early 1990s in a coastal area in the Gulf of Finland, coinciding with regime shifts in other areas of the Baltic Sea (Möllmann et al., 2005). The period was characterized by the introduction of the non‐native sediment‐dwelling polychaete genus *Marenzelleria* spp. to the system, and by the gradual decline and eventual disappearance of the amphipod *Pontoporeia femorata*, substantial decline of the amphipod *Monoporeia affinis,* and the simultaneous dramatic increase in the bivalve *Limecola balthica*. The present study was conducted in the coastal region of the western Gulf of Finland, which has experienced a 2°C temperature increase since the 1980s (Humborg et al., 2019), while the salinity has decreased during the 1980 and later stabilized (Rousi et al., 2013). There has also been a slight decrease in oxygen in the near‐bottom waters since 1979 (Rousi et al., 2013). Long‐term studies have indicated that zooplankton is influenced by changes in both salinity and temperature (Mäkinen et al., 2017; Suikkanen et al., 2013), and the survival of benthic taxa is tied to the prevailing oxygen conditions as their tolerances to low oxygen conditions vary (Villnäs et al., 2012).

The present study focuses on identifying and quantifying the biotic relationships between benthic and pelagic invertebrate taxa in a coastal area in the western Gulf of Finland using multivariate time‐series analysis, jointly modelling how biotic interactions and extrinsic variables are shaping the instantaneous population growth rates of the studied taxa. As many taxa have been shown to affect zooplankton benthic eggs in an experimental setting (Viitasalo, 2007), we expected the zoobenthic taxa to influence the annual abundances of both cladocerans and copepods. We especially expect amphipods and the bivalve *L. balthica* to negatively affect copepods and cladocerans due to their potential effect on emergence and hatching of nauplii from the sediment (Albertsson & Leonardsson, 2001; Viitasalo, 2007). We also consider extrinsic drivers and expect that oxygen will be the dominant extrinsic variable primarily influencing the benthic taxa, whereas temperature and salinity are assumed to be the most important ones for cladocerans and copepods. Finally, we investigate whether biotic interactions can be separated from abiotic effects on several taxa at community level.

## 2 MATERIAL AND METHODS

### 2.1 Study area and time series

#### 2.1.1 Biotic data

The zooplankton data have been gathered at Storfjärden in the western Gulf of Finland (59°50′N 23°15′E). The sampling was carried out using a 150 µm Hensen net hauled from 25 m to the surface, usually three times per month during 1966–1984 (Viitasalo et al., 1995), and once a month during 1993–2007 (newer data not available at the time). No comparable sampling was carried out between 1985 and 1993. The diameter of the net decreased from 0.72 m to 0.35 m between the two time periods, but the aim of the monitoring remained the same. All data have been enumerated in a similar fashion; each sample was split using a Folsom splitter into subsamples depending on the density of the sample (max 1/1024), and one to two subsamples per sample were counted. The number of individuals m^−3^ was calculated using the area of the net and the haul depth. Thus, the number of individuals is standardized to the size of the net, but caution should still be applied as the net area can affect capture efficiency. We restricted this study to the dominant calanoid copepods (hereafter copepods) and cladocerans in the area. The copepods include *Acartia* spp. and *Eurytemora affinis*, and the cladocerans mainly include *Bosmina* spp., as well as smaller numbers of *Chydorus* spp., and *Daphnia* spp. The copepod data include both adult and copepodite stages. We used zooplankton data from July, August, and September, as these months correspond to the timing of the benthic sampling. The chosen seasonal window also represents the period with highest abundances for the focal zooplankton taxa (Viitasalo et al., 1995).

The benthic data were gathered from August to October in the same area (59°85′N 23°27′E) as the zooplankton from a depth of 35 m using a Van Veen grab (1,115 cm^2^) (Rousi et al., 2013). The samples were sieved through a 1 mm mesh and subsequently counted and standardized to individuals m^−2^. Generally, there were triplicate grab‐samples each year, apart from two years with only one sample and five years with no sampling. The benthic taxa included in the analysis are *Marenzelleria* spp., other polychaetes (*Bylgides sarsi* and *Hediste diversicolor*), the bivalve *Limecola* (prev. *Macoma*) *balthica*, and amphipods (*Pontoporeia femorata* and *Monoporeia affinis*). These species generally constitute the bulk of the zoobenthic assemblages in the area (Rousi et al., 2013).

#### 2.1.2 Abiotic data

The environmental variables of interest are water temperature, salinity, and oxygen. Salinity and temperature data were provided by Tvärminne Zoological Station, University of Helsinki, (Finland) and the Finnish Meteorological Institute, and were primarily measured at discrete depths (0, 5, 10, 15, 20, and 30 m), with some additional CTD data used to supplement missing data. We used mean salinity and temperature values from 0 to 30 m to calculate time series of annual anomalies using a generalized additive mixed model with a cyclic spline for Julian day and a random intercept for each year (mgcv package in R; Wood, 2017). The oxygen level (O_2_ mg/L) was measured close to the sampling site (59°85′N, 23°26′E) from the bottom water layer at a depth of 33–34 m. We investigated linear trends in both the constructed anomalies and in the oxygen variable applying linear regression against year with AR (1) residuals, utilizing generalized least squares in the nlme package in R.

### 2.2 Statistical analyses

Several previous studies have used multivariate autoregressive models (MAR‐models) for quantifying interactions in plankton communities, simultaneously investigating the impact of extrinsic variables using time‐series data (Barraquand et al., 2018; Francis et al., 2012; Hampton et al., 2006; Ives et al., 2003). By utilizing time series of population abundances on the ln‐scale, these models correspond to a multispecies generalization of the Gompertz population model. We extend the MAR‐model, which includes process error only, to a multivariate state‐space model (SSM), which simultaneously accounts for both observation and process error (Durbin & Koopman, 2012). This is crucial as ignoring observation error in data severely biases (overestimates) the strength of negative density dependence (Knape & de Valpine, 2012) and is also known to bias interspecific interactions (Ives et al., 2003), both being of crucial interest in this study. Apart from the observation model, our model is similar to the log‐linear MAR‐models applied earlier, with a first order autoregressive relationship and a range of covariates.

The models were fit using the MARSS package, and all analyses were done in the R‐environment version 3.6.3 (Holmes et al., 2012; R Core Team, 2020). The abundances of the taxa are modelled as six underlying state variables in the process model (Equations 1 and 2) (*L. balthica*, polychaetes, *Marenzelleria* spp., amphipods, copepods, and cladocerans), arranged for each time step *t* in a vector (***x*** *_t_*). Each underlying state is influenced by nine (zooplankton) or three (benthos) ln‐transformed and mean centered observation time series (***y*** *_t_*; in total 30 time series) in the observation model (Equations 3 and 4). As the benthic time series included zeroes, we added one to all values in the benthic time series prior to taking the natural logarithm.

(1) $$x_{t}={\mathrm{Bx}}_{t‐1}+{\mathrm{Cc}}_{t}+w_{t}$$

(2) $$w_{t}∼N_{6}\left(0,Q\right)$$

(3) $$y_{t}=a+{\mathrm{Zx}}_{t}+v_{t}$$

(4) $$v_{t}∼N_{30}\left(0,R\right)$$

The states in the process model interact with each other as specified in the ***B*** matrix (6‐by‐6), where the diagonal contains the autoregressive coefficients (the density dependence), and the off‐diagonal elements (***B*** *_ij_;* *i* ≠ *j*) specify the effects of species’ *j* abundance on species’ *i* per capita growth rates. ***C*** is a 6‐by‐*k* matrix of environmental effect coefficients, and **c** *_t_* is a column vector with the *k* covariate values for time *t* (i.e., annual values of the environmental variables). The process error ***w*** *_t_* is assumed to have a multivariate normal distribution, with a full variance–covariance matrix ***Q*** whose elements were freely estimated (Equation 2) and represents the unexplained process variance, which is typically interpreted as unexplained environmental effects. The investigated state variables (Equation 1) are linked to the observation times series (Equation 3) through the ***Z*** matrix (30‐by‐6) and scaled with vector ***a***. As the zooplankton abundance levels were expected to vary during the different months, ***a*** enabled different intercepts. For the benthic taxa, ***a*** was set to 0. Also, the observation error (***v*** *_t_*) is assumed to have a multivariate normal distribution, but with a diagonal variance–covariance matrix ***R***. Each benthic state has its own estimated observation error term, except for the zooplankton that have their observation error variances fixed at ***R***_13,13_ to ***R***_22,22_ = 0.259 for the copepods and ***R***_23,23_ to ***R***_30,30_ = 0.413 for the cladocerans (Equation 4). The initial values of the state variables (***x***_1_) were estimates as parameters in the model.

Since the zooplankton time series has no replicates (single hauls were used throughout the sampling period, as is the standard in pelagic sampling: HELCOM 2014), we used data from a field study conducted in 2016 to estimate the zooplankton observation error variances. Estimating and fixing the observation error a priori will facilitate the estimation of the process error in our community model for taxa with an unreplicated time series, as the observation and process errors are notoriously difficult to partition (Dennis et al., 2010). Planktonic samples for the observation error study were collected at three nearby locations in June and August 2016 in triplicate, resulting in 30 net tows (detailed description in the Appendix S1). Using the field study that provided replicated data we estimated the error variance separately for copepods and cladocerans using linear models (extracting the estimated residual variance), with the ln‐abundance as response, and sampling month, station, and their interaction as explanatory variables. The estimated residual variances from the copepod and cladoceran linear models were 0.259 and 0.413, respectively.

To investigate the community interactions and to find out whether we can detect benthic–pelagic interactions on interannual scale, we consider four alternative scenarios for the community interactions: one full model including plausible interspecific interactions, a simplified version considering only the benthic–pelagic interactions (BPC only), one considering no benthic–pelagic interactions and only interactions within the benthic taxa (no BPC), and one with no interactions whatsoever (no interactions). The interactions in the full model were based on species interactions found in literature, including competition, and predation (Table 1). To estimate the biotic interactions between the taxa we use the species interaction matrix (***B***) for defining our four scenarios, fixing predefined elements to zero (Figure S1). As *Marenzelleria* spp. appeared in the samples for the first time in 1991, its interactions with other taxa were set to zero prior to this time. Also, the environmental covariates were considered from 1990 for *Marenzelleria* spp. by setting all coefficients in row three (corresponding to the covariate effects on *Marenzelleria* spp.) of ***C*** to zero prior to the species’ establishment in the area.

**TABLE 1:** A summary of the interspecific interactions between the studied taxa as outlined in literature from the Baltic Sea

Taxon	Affected taxa	Interaction type	Expected effect	Reference
*L. balthica*	Amphipoda	Resource competition	−	Karlson et al. (2010, 2015)
	Cladocerans, Copepods	Predation on benthic eggs	−	Karlson and Viitasalo‐Frösen (2009)
Polychaeta (*H. diversicolor* & *B. sarsi*)	*L. balthica* larvae, Amphipoda	Predation	−	Rönn et al. (1988), Sarvala (1971)
*Marenzelleria* spp.	Cladocerans, Copepods	No effect on eggs through bioturbation	0	Viitasalo (2007)
Amphipoda	*L. balthica*	Predation on larvae, competition	−	Elmgren et al. (1986)
	Cladocerans, Copepods	Reduced hatching of eggs	−	Viitasalo et al. (2007), Viitasalo (2007)

Note

The table details the effects of each relevant taxon (Taxon) on other taxa (Affected taxa), the nature of the relationship (Interaction type), the expected sign of the interaction coefficient (Expected effect; positive +, negative −, or neutral 0), and references to the literature (Reference).

**FIGURE 1:**
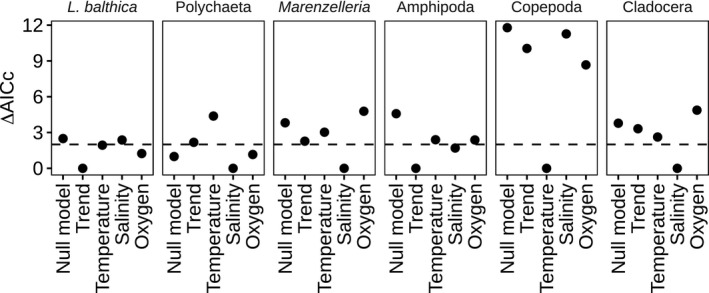
Comparison of differences in AICc (ΔAICc) compared with the most parsimonious model for the four univariate candidate SSMs used for choosing environmental covariates. The horizontal line indicates an AICc difference of 2. The null model includes no trend, whereas all other models do in addition to the environmental covariate

All community models were additionally investigated including or excluding environmental covariates, leading to a total of eight models (four interaction scenarios with or without environmental covariates). Additionally, all models always included year as a covariate to account for long‐term population change. To avoid over‐parameterization, environmental covariates were included in taxon‐specific full models based on preliminary analyses. For this purpose, we used the above mentioned SSM approach but for one taxon at a time. The models in both the preliminary and main analysis were compared using model selection with Akaike information criterion corrected for small sample size (AICc). The models included either no covariates as a null model, only a trend or a trend and one environmental covariate. All covariates were standardized to zero mean and divided by their standard deviation (z‐scored). The environmental variables considered are annual mean salinity and temperature anomalies and late summer bottom water oxygen. We identified the model with the lowest AICc score for each taxon and included its covariates in the community SSM for the respective taxon (Figure 1). For *Marenzelleria* spp., the other polychaetes and the cladocerans, salinity was selected as the environmental covariate (in addition to the trend), whereas copepods, included temperature, and *Limecola balthica* and the amphipods included only the temporal trend.

The model is flexible with regard to certain nonstationary properties. First, as year is included as a covariate, the model assumes a trend‐stationary process on the log‐scale, that is, the process is stationary apart from a log‐linear trend. Secondly, estimation of the initial values of the state variables allows for transient dynamics. Hence, the model can describe, for example, a newly established population (e.g., *Marenzelleria* spp.) growing from low abundance, before reaching a level where it starts to fluctuate in a stationary manner.

All models were fit using the EM algorithm (Holmes, 2013). Standard errors (*SE*) and confidence intervals (CI) for the parameter estimates for the most parsimonious model were estimated using parametric bootstrap with 1,000 resampling events. We consider 95% and 90% CI of covariate estimates and community interactions that do not cross zero to indicate statistically significant effects and tendencies, respectively. Model checking was conducted by visually inspecting quantile–quantile plots of the residuals and graphs of residuals regressed against fitted values from both the observation and process model, as well as graphs of the autocorrelation functions of the process residuals.

## 3 RESULTS

**FIGURE 2:**
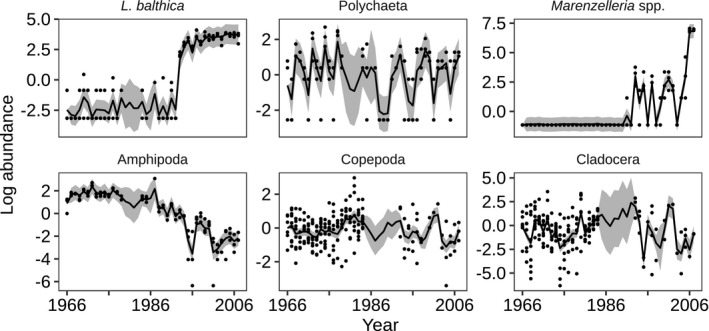
Estimated mean centered state variables (black line) with 95% CI (grey area) for the most parsimonious community models with no BPC but including environmental variables. Black points are raw data. For the cladocerans and copepods, the raw data have been adjusted according to the scaling parameter **a** in the observation model (Equation 4)

Based on the estimated underlying states, there was a sudden increase in *L. balthica* corresponding to the timing of the introduction of *Marenzelleria* spp. in the early 1990s, while the amphipods display a more gradual decline over the whole period (Figure 2). The cladocerans and copepods fluctuate around a steady state, whereas the other polychaetes (*H. diversicolor* and *B. sarsi*) displayed somewhat cyclic patterns. Long‐term changes were also prevalent in the abiotic data (Figure S2). There was an upward trend in the annual water temperature (0–30 m), with a linear annual increase of 0.04°C (*SE* 0.010). The annual salinity (0–30 m) displayed a more complicated pattern, reaching its maximum values, approximately 6.7 in the late 1970s. The overall linear annual trend, however, was slightly negative: 0.009 (*SE* 0.004). The oxygen in the bottom water decreased by 0.047 mg/L (*SE* 0.020) annually over the study period.

**FIGURE 3:**
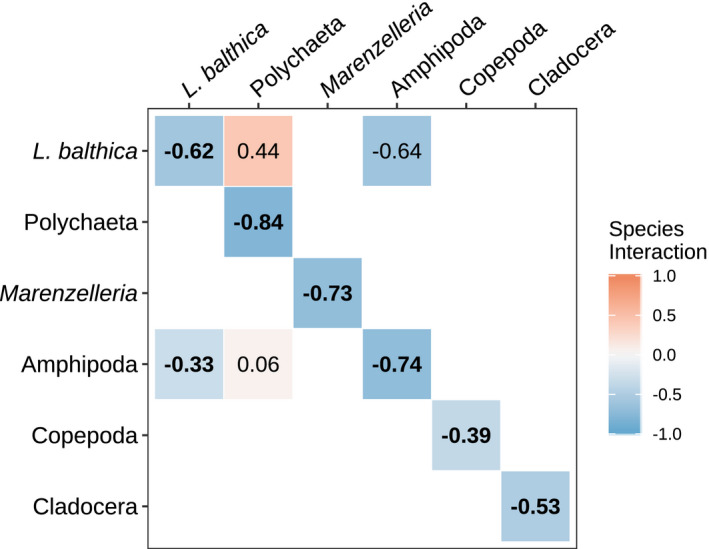
Community interactions in the most parsimonious model including the environmental covariates and no benthic–pelagic coupling. The matrix is interpreted as the effect of the taxa in the columns on the taxa in the rows. Bolded values off the diagonal indicate interactions where the 90% CI exclude 0. As the single estimated interactions often crossed 0, we applied 90% confidence intervals, to highlight which interactions were contributing most likely to the dynamics of the system. The strength of the species interaction is indicated by the color and squares with no value were not considered. To make the interpretation of the density dependence on the diagonal comparable to the off‐diagonal interaction terms, the identity matrix was subtracted from the matrix

In the model comparison between the four interaction scenarios, the best performing model included interactions between only the benthic species. Hence, contrary to our expectations, it included no interactions between the benthic and pelagic taxa (Table 2 and Figure S3). Among the interactions between the benthic taxa included in the most parsimonious model, only one of the interactions had a 90% confidence interval excluding zero (Figure 3). There was a negative effect of *L. balthica* on amphipods, which was in accordance with our expectation. Notably, also the estimated reciprocal effect of amphipods on *L. balthica* was negative (indicating competition), and almost twice as large, but it also had larger uncertainty and the 90% CI included zero. For all other estimated interspecific interactions, the 90% CI included zero (Table 2). Density dependence was present (diagonal of ***B*** < 1) in all investigated taxa, and the magnitude within each taxon was consistent between all investigated models. The values range from 0.164 to 0.608 in the most parsimonious model. Both the graphs of the process‐ and observation model residuals looked reasonable (Figures [Link], [Link], [Link]). The models that included environmental covariates always performed better (ca 10 units AICc) compared with their counterpart without environmental covariates, and the direction of the biotic interactions remained the same (Figure S3, Table 2).

**TABLE 2:** The eight investigated community models (*n* = 841 for all) together with the number of estimated parameters (*k*), log likelihood (LogLik), Akaike information criterion corrected for small sample size (AICc), and difference in AICc compared with the most parsimonious model (ΔAICc)

Model	*k*	LogLik	AICc	ΔAICc
No BPC + Env.	55	−1,682.8	3,483.5	0
No interactions + Env.	51	−1,691.2	3,491.2	7.7
All interactions + Env.	61	−1,680.0	3,491.8	8.2
No BPC	51	−1,692.2	3,493.1	9.5
Only BPC + Env.	57	−1,687.8	3,498.0	14.5
No interactions	47	−1,700.7	3,501.2	17.6
All interactions	57	−1,689.4	3,501.3	17.7
Only BPC	53	−1,697.2	3,507.8	24.2

Note

The models are presented in ascending order according to ΔAICc, starting with the most parsimonious one. BPC indicates benthic–pelagic coupling and Env. models where environmental variables are included.

**FIGURE 4:**
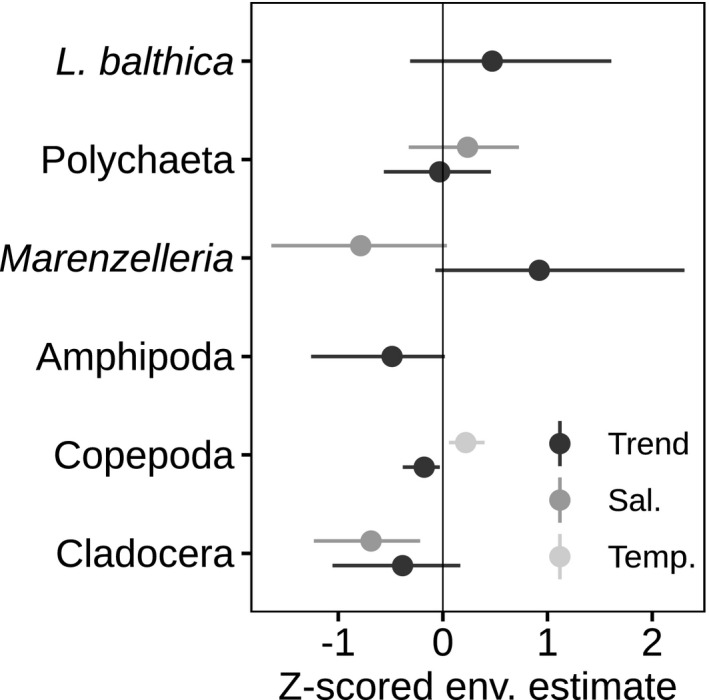
The effects of the environmental covariates and their 95% CI in the most parsimonious community model with no benthic–pelagic coupling

Contrary to our expectation, oxygen was not included in any of the most parsimonious models. In the most parsimonious community model, salinity was included for *Marenzelleria* spp., other polychaetes, and for cladocerans, but the 95% CI did not include zero only for cladocerans. Temperature was only included for the copepods and influenced them positively. Both copepods and amphipods had a negative partial trend (Figure 4). The estimates of the environmental effects in the community model correspond well with the results of the preliminary taxon‐wise covariate investigation, but with higher uncertainty in the estimates (Figure 4 and Figure S4).

All estimated model parameters for the most parsimonious model are presented in Table S1. *Marenzelleria* spp. had the highest estimated process error (***Q***_3,3_ = 2.17: Table S2). The residual correlations (***Q*** matrix) between taxa were relatively weak except for the correlation of 0.69 between cladocerans and copepods, which was the only correlation where the 95% CI did not cross zero (Table S3). The correlations were calculated using the process error covariance matrix and the CI from that matrix is used to also indicate significance for the correlations (Table S2). The environmental correlation within the zoobenthos taxa generally had positive signs (0.09 to 0.43), and the correlation between zoobenthos and zooplankton generally had negative signs (−0.42 to −0.05), with the exception of the correlation between *Marenzelleria* spp. and copepods (0.17).

## 4 DISCUSSION

### 4.1 Detecting no coupling

Contrary to our expectations the most parsimonious model did not include interactions between benthic and planktonic taxa. This is unexpected, as laboratory studies have suggested that benthic taxa can exert substantial predation pressure on benthic eggs of both copepods and cladocerans (Karlson & Viitasalo‐Frösen, 2009; Viitasalo, 2007). It is possible that the zooplankton population is more limited by the availability of resources in the area than by regulation from the benthos on their recruitment from benthic eggs. The amounts of zooplankton eggs in the surface of the sediment have been shown to vary over the season and the development time of the eggs is affected by prevailing temperature (Katajisto et al., 1998); hence, predation effects on eggs would likely be challenging to detect in the pelagic population. Biotic interactions between zooplankton and benthic taxa might thus be better detectable on shorter time scale or during a specific seasonal window.

The observed high correlation between copepods and cladocerans in the ***Q*** matrix indicates that there was unexplained correlated variation between these taxa. This correlation among competitors can arise from a correlated environment (Ripa & Ives, 2003, 2007), and as phytoplankton was not included in the study it could well reflect food availability. Egg production of the main copepod taxon *Acartia bifilosa* is known to be influenced by food availability (Koski & Kuosa, 1999). None of the benthic taxa had as strong positive residual correlations as the zooplankton (Table S3), even though *M. affinis* is known to depend on fresh sedimentary material (Eriksson Wiklund & Andersson, 2014). Benthic taxa are more dependent on material sedimentated during the spring bloom (Elmgren, 1978; Uitto & Sarvala, 1991), and the investigated zooplankton abundances in the present study were all measured during late summers. Another explanation for the correlation between the zooplankton groups is predation, as the zooplankton community experiences heavy predation in late summer (Hansson et al., 1990), and as also a shared predator can give rise to temporal synchrony (Huber & Gaedke, 2006). Overall, the process correlations between zooplankton and zoobenthos were uncertain but with negative signs, which suggest that the two benthic and pelagic taxa are inversely affected by the environment and it can for example reflect within season competition on resources, as more efficient pelagic processes lead to decreased sedimentation and thus less organic matter for benthos (Tamelander et al., 2017). Whenever the goal is to investigate only the interactions between benthic and pelagic species, simplifying the model to a two‐state model can be a fruitful option.

All taxa displayed relatively strong density dependence, which traditionally would suggest a high level of intraspecific competition. Some studies have indeed concluded that benthic taxa such as *L. balthica* and *Marenzelleria arctia* experience intraspecific density dependence as a result of competition for space and food limitation (Ehrnsten et al., 2019; Karlson et al., 2011; Kauppi et al., 2018), which may in fact override the direct links between the different components, that is, the benthic and pelagic assemblages. For shorter lived taxa like zooplankton, it is more likely that the density dependence is a statistical property of the time series, whereas for the more long‐lived benthos the effects could reflect ecological effects. Some benthic taxa like *L. balthica* can live up to 30 years (Segerstråle, 1960). Whereas competition for food resources is likely a fact in both zones, relevant competition for space is likely to occur only for the benthic taxa.

### 4.2 Biotic and abiotic drivers

The detected links in the benthic community observed between *L. balthica* and the amphipods are well supported by the literature and early field investigations by Segerstråle (1962, 1978) noted an inverse relationship between *L. balthica* and *M. affinis*. More recent studies suggest that the taxa utilize similar resources because their isotopic niches overlap (Karlson et al., 2015), although *L. balthica* is at a lower trophic level compared with the amphipods in the Gulf of Finland (Kiljunen et al., 2020). *L. balthica* is known to show high plasticity in its feeding behavior (Olafsson, 1986; Törnroos et al., 2015), explaining the low trophic position in this system. The amphipod *M. affinis* is also known to directly prey on *L. balthica* larval stages influencing recruitment of young individuals to the sediment (Elmgren et al., 1986). Whereas we did not investigate the biomass of the taxa, Rousi et al. (2013) reported an increase in individual biomass of *L. balthica*. It is possible that the decrease in numbers of amphipods, has led to decreased competitive interference between the groups, enabling bigger size for *L. balthica*. Elmgren et al. (1986) suggested that the presence of *M. affinis* decreases the growth rate of *L. balthica* by small nonlethal disturbances such as bioturbation and Karlson et al. (2010), Karlson et al. (2011) have experimentally shown competitive interference. Thus, the decline in amphipods can also have affected growth rates. Whereas our results clearly indicate that the model including the interactions was the superior one in the investigated subset, it is not possible to conclusively state the direction of the effect, considering the broad CI.

When investigating environmental effects on food webs, not accounting for environmental correlation between taxa can confound the identification of species interactions (Ripa & Ives, 2003, 2007). It was clear that the models including the environment were more parsimonious, compared with their non‐environment counterparts, but the interaction strengths and directions were similar in both groups of models. The effects of the environmental variables were more distinguishable in the zooplankton groups. The negative salinity effect seems reasonable as the taxa are documented to benefit from lower salinity (Kuosa et al., 2017). The positive temperature effect on the copepods is also in line with tolerances of the most abundant species, *Acartia bifilosa*, which has a broad temperature tolerance, with suboptimal temperatures above 24°C (Koski & Kuosa, 1999). Based on the results of the present study, an increase in temperature could benefit copepods in terms of abundance.

The fact that none of the best performing single‐species models for the benthic species included oxygen, likely has more to do with the suitability of the variable. The measurements were taken from the water close to the bottom and not from the sediment. Additionally, a snapshot with oxygen measurements for late summer only might not reflect the overall oxygen conditions for the entire year well enough, as oxygen conditions can vary considerably, both seasonally and spatially (Virtanen et al., 2019). Also, a temporary drop in the oxygen level can be detrimental for sensitive taxa, such as *M. affinis* that feed less in hypoxic conditions (Ejdung et al., 2008). In contrast, both *L. balthica* and *Marenzelleria* spp. are more tolerant to hypoxia (Norkko et al., 2012; Villnäs et al., 2012).

### 4.3 Potential shifts in interactions

Interactions within communities can vary and change over time because of temporally variable climate effects (Francis et al., 2012). In extreme cases, if a tipping point is reached, long‐term changes in one direction can lead to ecological regime shifts (Casini et al., 2009; Collie et al., 2004). Studies have shown that biotic interactions can fluctuate with changing environmental conditions and species composition (Francis et al., 2012, 2014). Previous work suggests that *Marenzelleria* spp., which at the station presumably is *M. arctia,* has colonized an empty niche (Karlson et al., 2015; Norkko et al., 2012). Regardless, it is apparent that a substantial shift occurred in the community during the 1990s (Rousi et al., 2013). This type of shifts, whether due to changes in community composition or abiotic factors, can also induce changes in biotic interactions. The interactions and environmental impacts reported in this study are averages over the entire period, so potential shifts in interactions would be averaged out. Ideally, we would have compared the situation before and after the introduction of *Marenzelleria* spp., but due to the unfortunate fact that it coincides with a gap in the zooplankton time series, this approach was not possible. Changes in phenology are also suggested to influence the strength of food web interactions (Francis et al., 2014), for example, through the induction of a within season temporal mismatch between taxa (Cushing, 1969, 1990). There are indications of changes in the timing of both the phytoplankton spring bloom and zooplankton emergence in the Baltic Sea (Hjerne et al., 2019; Klais et al., 2017). Both the averaging of the biotic interactions and the potential phenology changes could have contributed to the fact that the CIs were so broad.

## 5 CONCLUSIONS

Time series are crucial for investigating long‐term population changes. Although we were not able to detect any benthic–pelagic coupling in the form of clear interactions between zoobenthos and zooplankton, the most parsimonious model indicated that biotic interactions within the benthic community are important. The shorter generation time of zooplankton compared with benthic taxa potentially contributed to the fact that we were unable to detect interactions between zooplankters and benthic species at the focal time‐scale. The competitive interference tendency of *L. balthica* on amphipods has likely contributed to the observed decrease in amphipods over time. We also detected positive within season correlations between copepods and cladocerans, which are most likely a reflection of the availability of phytoplankton.

## CONFLICT OF INTEREST

None declared.

## AUTHOR CONTRIBUTION

**Louise Forsblom:** Conceptualization (equal); Data curation (equal); Formal analysis (lead); Funding acquisition (equal); Investigation (equal); Methodology (equal); Validation (lead); Visualization (lead); Writing‐original draft (lead); Writing‐review & editing (equal). **Andreas Lindén:** Conceptualization (equal); Formal analysis (supporting); Funding acquisition (equal); Methodology (equal); Supervision (equal); Validation (supporting); Visualization (supporting); Writing‐original draft (supporting); Writing‐review & editing (equal). **Jonna Engström‐Öst:** Conceptualization (equal); Funding acquisition (equal); Investigation (equal); Resources (lead); Supervision (equal); Visualization (supporting); Writing‐original draft (supporting); Writing‐review & editing (equal). **Maiju Lehtiniemi:** Conceptualization (equal); Data curation (equal); Methodology (supporting); Resources (supporting); Visualization (supporting); Writing‐original draft (supporting); Writing‐review & editing (equal). **Erik Bonsdorff:** Conceptualization (equal); Funding acquisition (equal); Methodology (supporting); Supervision (equal); Visualization (supporting); Writing‐original draft (supporting); Writing‐review & editing (equal).

## Supporting information

Figure S2

Figure S3

Figure S4

Figure S5

Figure S6

Figure S7

Appendix S1

## Data Availability

The field data from 2016 and the input for the community model: https://doi.org/10.5061/dryad.4b8gthtbs.
