# Supplementary material for: Identifying biotic drivers of population dynamics in a benthic–pelagic community
Source: Ecol Evol. 2021 Mar 28;11(9):4035–45. doi: 10.1002/ece3.7298 (PMC8093679; doi:10.1002/ece3.7298)
Supplement: Supplementary file 7 — Appendix S1 [file ECE3-11-4035-s002.pdf]

Appendix S1-7

Field sampling to determine the observation error variance in cladocerans and copepods

To fix the sampling error a field study was conducted during 2016, where zooplankton was sampled from 25 m to the surface using a 150 µm Hensen net. The enumeration of cladocerans and calanoid copepods was conducted in accordance with the time-series data. Three replicate net-samples were taken during two days in June and one day in August at three stations, and additionally at one of the stations a second day in August leading to a total of 30 samples (https://doi.org/10.5061/dryad.4b8gthtbs). Using the data from the field study and fitting linear models with the combination of sampling station and sampling month, and their interaction as a factor variable, we estimated residual variances of 0.259 and 0.413, for the copepods and cladocerans, respectively. These values are higher compared to the observation error variance of 0.16 used by Ives et al. (2003) for zooplankton in a lake system. To investigate the sensitivity of the observation error estimate we compared our value to that of Ives et al. (2003). When we ran the best fit model using an observation error variance of 0.16 for the zooplankton, the results were qualitatively the same as with our estimated error variances, but with narrower confidence intervals for the estimated parameters in the interaction matrix ***B***.

Table S1. Parameter estimates (Estimate), standard errors (SE) and 95% confidence intervals (lwr CI, upr CI) for the most parsimonious model, with no benthic–pelagic coupling and with the environmental variables included. The letters in the parameter names correspond to Equations 1–4.

Parameter	Estimate	SE	lwr CI	upr CI
a2.copepoda	0.126	0.085	−0.031	0.291
a3.copepoda	0.143	0.084	−0.018	0.315
a2.cladocera	1.277	0.116	1.033	1.491
a3.cladocera	0.400	0.118	0.167	0.619
r.limecola	0.721	0.124	0.490	0.981
r.polychaeta	1.085	0.191	0.738	1.514
r.maren	0.296	0.045	0.217	0.388
r.amphi	0.509	0.086	0.352	0.685
b.limecola.limecola	0.384	0.222	−0.139	0.769
b.lim.amph	−0.329	0.180	−0.676	0.057
b.pol.lim	0.435	0.324	−0.132	1.185
b.polychaeta.polychaeta	0.164	0.231	−0.322	0.578
b.pol.amph	0.056	0.227	−0.465	0.504
b.amph.lim	−0.641	0.396	−1.500	0.081
b.amphipoda.amphipoda	0.258	0.264	−0.237	0.820
b.copepoda.copepoda	0.608	0.158	0.197	0.832
b.cladocera.cladocera	0.474	0.153	0.100	0.708
b.marenzelleria.marenzelleria	0.272	0.243	−0.308	0.655
q.limecola.limecola.	0.842	0.290	0.239	1.348
q.polychaeta.limecola.	0.176	0.269	−0.356	0.723
q.marenzelleria.limecola.	0.414	0.325	−0.166	1.092
q.amphipoda.limecola.	0.059	0.161	−0.243	0.408
q.copepoda.limecola.	−0.041	0.102	−0.247	0.161
q.cladocera.limecola.	−0.465	0.354	−1.190	0.253
q.polychaeta.polychaeta.	1.348	0.438	0.497	2.187
q.marenzelleria.polychaeta.	0.437	0.367	−0.246	1.213
q.amphipoda.polychaeta.	0.353	0.210	−0.043	0.770
q.copepoda.polychaeta.	−0.146	0.124	−0.403	0.102
q.cladocera.polychaeta.	−0.081	0.380	−0.861	0.662
q.marenzelleria.marenzelleria.	2.167	0.501	1.181	3.109
q.amphipoda.marenzelleria.	0.166	0.245	−0.323	0.655
q.copepoda.marenzelleria.	0.097	0.135	−0.158	0.389
q.cladocera.marenzelleria.	−0.239	0.421	−1.065	0.596
q.amphipoda.amphipoda.	0.497	0.162	0.129	0.748
q.copepoda.amphipoda.	−0.116	0.079	−0.264	0.051
q.cladocera.amphipoda.	−0.140	0.261	−0.693	0.379
q.copepoda.copepoda.	0.154	0.054	0.055	0.266
q.cladocera.copepoda.	0.382	0.143	0.135	0.688
q.cladocera.cladocera.	1.992	0.558	0.945	3.077
x0.limecola	−2.479	0.475	−3.373	−1.525
x0.polychaeta	−0.603	0.603	−1.749	0.590
x0.maren	−1.151	0.313	−1.737	−0.535
x0.amphi	0.880	0.406	0.086	1.650
x0.copepoda	−0.007	0.173	−0.364	0.314
x0.cladocera	−0.127	0.224	−0.559	0.299
c.limecola.trend	0.471	0.475	−0.313	1.609
c.polychaeta.trend	−0.031	0.254	−0.564	0.459
c.amphipoda.trend	−0.486	0.326	−1.258	0.018

c.copepoda.trend	−0.179	0.091	−0.382	−0.030
c.cladocera.trend	−0.385	0.299	−1.054	0.166
c.polychaeta.sal	0.236	0.276	−0.326	0.726
c.cladocera.sal	−0.686	0.255	−1.232	−0.218
c.copepoda.temp	0.218	0.086	0.058	0.398
c.marenzelleria.trend	0.921	0.619	−0.072	2.308
c.marenzelleria.sal	−0.785	0.438	−1.640	0.038

Table S2 The variance–covariance matrix of the process error (***Q***) for the most parsimonious model excluding BPC. Bold values indicate parameters for which the 95% CI do not cross zero.

	L. balthica	Polychaeta	*Marenzelleria*	Amphipoda	Copepoda	Cladocera
L. balthica	**0.842**	0.176	0.414	0.059	−0.041	−0.465
Polychaeta	0.176	**1.348**	0.437	0.353	−0.146	−0.081
Marenzelleria	0.414	0.437	**2.167**	0.166	0.097	−0.239
Amphipoda	0.059	0.353	0.166	**0.497**	−0.116	−0.140
Copepoda	−0.041	−0.146	0.097	−0.116	**0.154**	**0.382**
Cladocera	−0.465	−0.081	−0.239	−0.140	**0.382**	**1.992**

Table S3 The correlation matrix of the process error (***Q***) for the most parsimonious model excluding BPC. Bold values indicate off-diagonal correlations for which the 95% CI do not cross zero.

	L. balthica	Polychaeta	*Marenzelleria*	Amphipoda	Copepoda	Cladocera
L. balthica	1	0.166	0.306	0.091	−0.114	−0.359
Polychaeta	0.166	1	0.255	0.431	−0.320	−0.050
Marenzelleria	0.306	0.255	1	0.160	0.167	−0.115
Amphipoda	0.091	0.431	0.160	1	−0.419	−0.140
Copepoda	−0.114	−0.320	0.167	−0.419	1	**0.690**
Cladocera	−0.359	−0.050	−0.115	−0.140	**0.690**	1

$$\begin{bmatrix} b_{LimLim} & b_{PolLim} & 0 & b_{AmpLim} & 0 & 0 \\ 0 & B_{PolPol} & 0 & 0 & 0 & 0 \\ 0 & 0 & b_{MarMar} & 0 & 0 & 0 \\ b_{LimAmp} & b_{PolAmp} & 0 & b_{AmpAmp} & 0 & 0 \\ b_{LimCop} & 0 & b_{MarCop} & b_{AmpCop} & b_{CopCop} & 0 \\ b_{LimCla} & 0 & b_{MarCla} & b_{AmpCla} & 0 & b_{ClaCla} \end{bmatrix}$$

$$\begin{bmatrix} b_{LimLim} & 0 & 0 & 0 & 0 & 0 \\ 0 & B_{PolPol} & 0 & 0 & 0 & 0 \\ 0 & 0 & b_{MarMar} & 0 & 0 & 0 \\ 0 & 0 & 0 & b_{AmpAmp} & 0 & 0 \\ b_{LimCop} & 0 & b_{MarCop} & b_{AmpCop} & b_{CopCop} & 0 \\ b_{LimCla} & 0 & b_{MarCla} & b_{AmpCla} & 0 & b_{ClaCla} \end{bmatrix}$$

$$\begin{bmatrix} b_{LimLim} & b_{PolLim} & 0 & b_{AmpLim} & 0 & 0 \\ 0 & B_{PolPol} & 0 & 0 & 0 & 0 \\ 0 & 0 & b_{MarMar} & 0 & 0 & 0 \\ b_{LimAmp} & b_{PolAmp} & 0 & b_{AmpAmp} & 0 & 0 \\ 0 & 0 & 0 & 0 & b_{CopCop} & 0 \\ 0 & 0 & 0 & 0 & 0 & b_{ClaC} \end{bmatrix}$$

$$\begin{bmatrix} b_{LimLim} & 0 & 0 & 0 & 0 & 0 \\ 0 & B_{PolPol} & 0 & 0 & 0 & 0 \\ 0 & 0 & b_{MarMar} & 0 & 0 & 0 \\ 0 & 0 & 0 & b_{AmpAmp} & 0 & 0 \\ 0 & 0 & 0 & 0 & b_{CopCop} & 0 \\ 0 & 0 & 0 & 0 & 0 & b_{ClaCla} \end{bmatrix}$$

Equation S1. Interaction matrices ***B*** for the different scenarios of benthic–pelagic interactions. From top to bottom: full literature based, only benthic pelagic interactions, no benthic pelagic interactions and no interactions.

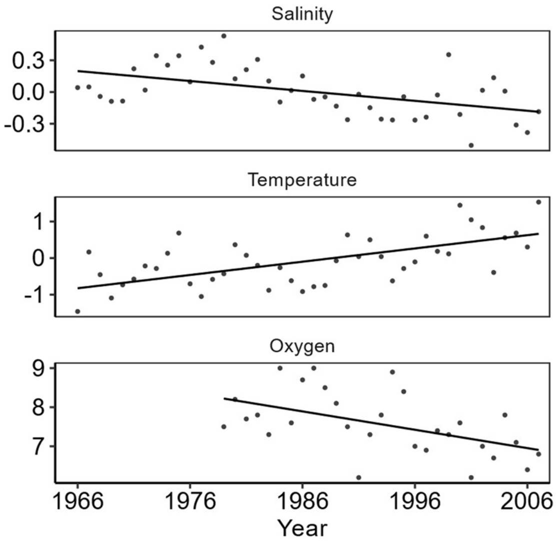

Figure S2. Annual salinity and temperature (°C) anomalies at 0–30 m depth and bottom water oxygen concentration (mg l^{-1}), illustrated as (dots) and fitted linear temporal trends (black lines).

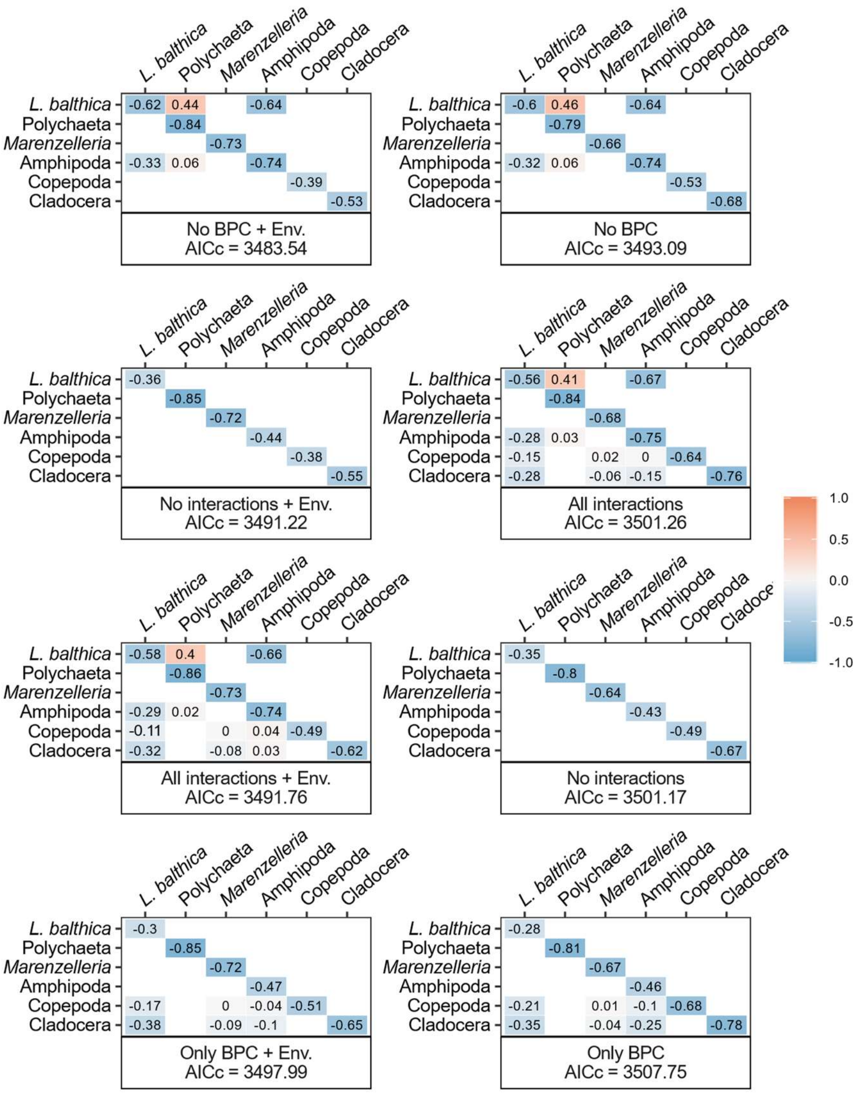

Figure S3. The community interactions for the four scenarios with environmental covariates included (panels in the left column) and without covariates included (panels in the right column). The models are sorted according to AICc values within the column, starting with the most parsimonious models from the top. The effects in each panel are interpreted as the effect of the taxa in the columns on the taxa in the rows of the matrix (e.g. in the most parsimonious model upper left, the estimated effect of Polychaeta on *L. balthica* is 0.44). The strength of the species interaction is indicated by the colour and interactions represented by combinations of row and columns without a value were not considered. To make the interpretation of the density dependence on the diagonal comparable to the interaction terms off the diagonal, the identity matrix was subtracted from each matrix.

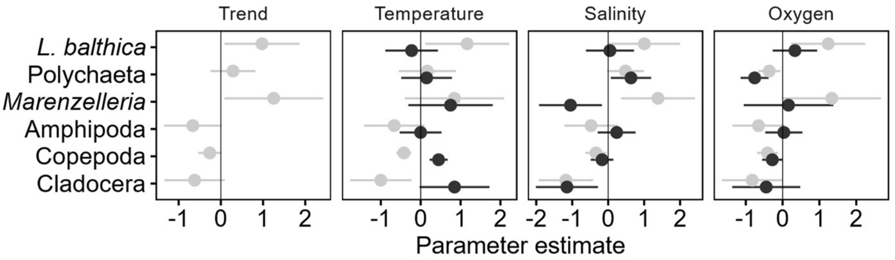

Figure S4. The estimated effects (filled circles) of the extrinsic variables and their 95 % confidence intervals (horizontal lines) from the preliminary covariate selection. Grey indicates the temporal trend (left panel) and partial temporal trends (the rest), while black indicates the z-scored effects of oxygen in late summer (mg l^{-1}), annual salinity and annual temperature anomalies (°C).

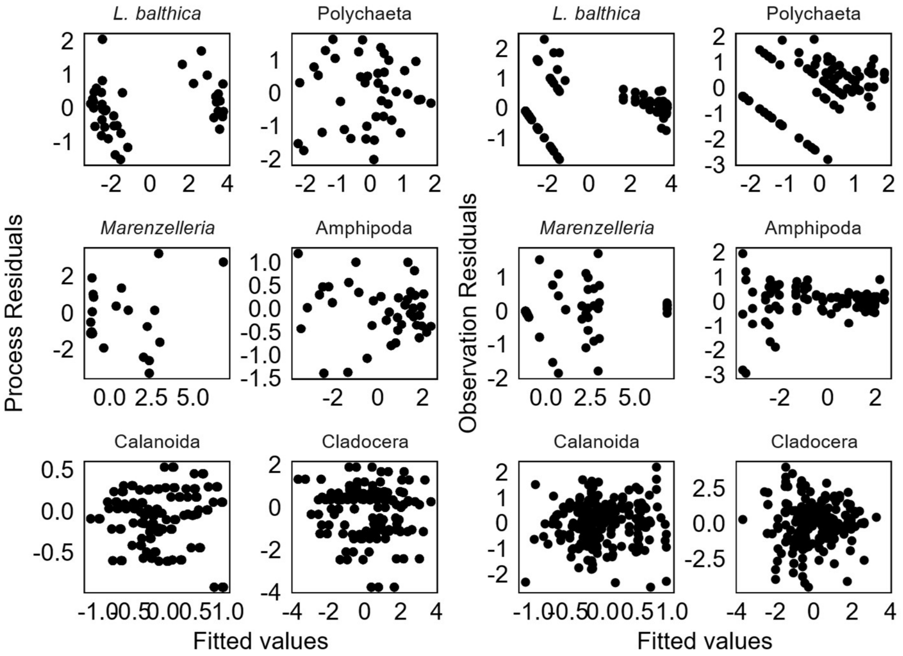

Figure S5. Process residuals (two leftmost columns) and observation residuals (two rightmost columns) plotted against the fitted values. The NA values have been omitted from the observation residuals.

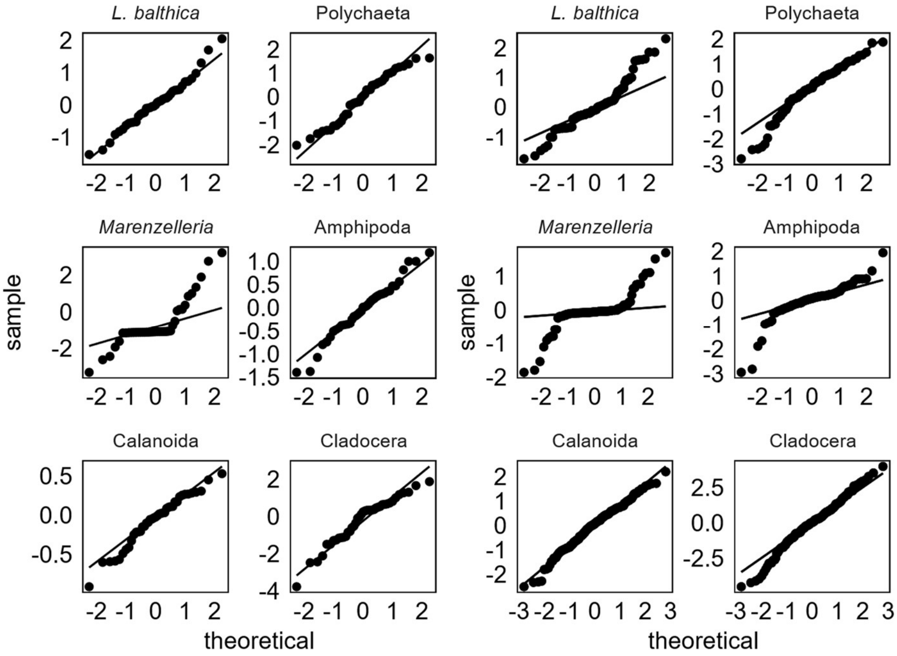

Figure S6. Quantile–quantile graphs of the process residuals (two leftmost columns) and observation residuals (two rightmost columns). The NA values have been omitted from the observation residuals.

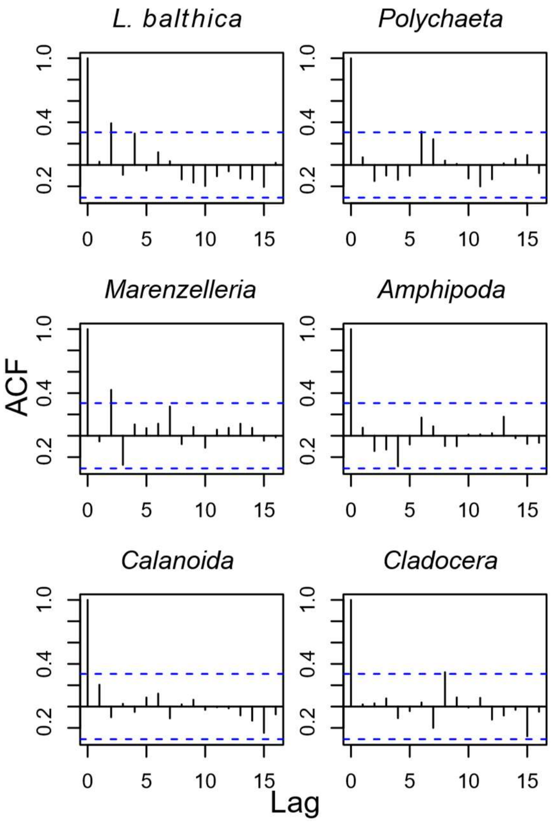

Figure S7. Autocorrelation functions of the process residuals.
